# Comparison of Insulin Detemir and Insulin Glargine for Hospitalized Patients on a Basal-Bolus Protocol

**DOI:** 10.3390/pharmacy5020022

**Published:** 2017-04-23

**Authors:** Sondra Davis, Chad Friece, Nicki Roderman, Darrell Newcomer, Evangelina Castaneda

**Affiliations:** 1 Medical Center Arlington, 3301 Matlock Road, Arlington, TX 76015, USA; darrell.newcomer@hcahealthcare.com; 2 The Medical Center of Plano, 3901 West 15th Street, Plano, TX 75075, USA; chad.friece@hcahealthcare.com (C.F.); evanca75@gmail.com (E.C.); 3 Denton Regional Medical Center, 3535 S Interstate 35 E, Denton, TX 76210, USA; nicki.roderman@hcahealthcare.com

**Keywords:** basal-bolus, insulin detemir, insulin glargine, long-acting insulin, inpatient, hospital, acute care

## Abstract

**BACKGROUND:** The primary purpose of this study is to determine whether insulin detemir is equivalent to insulin glargine in controlling hyperglycemia for the adult hospitalized patient on a basal-bolus treatment regimen. **METHODS:** A retrospective study was conducted at two acute care hospitals within the same health system. Patients from both facilities who were initiated on a basal-bolus subcutaneous insulin regimen were included in the study. The basal-bolus regimen consisted of three components: basal, bolus, and corrective insulin with only the data from the first seven days analyzed. Once the basal-bolus protocol was initiated, all previous glycemic agents were discontinued. The target glycemic goal of the study was 100–180 mg/dL. **RESULTS**: In both groups, 50% of the patients had achieved the target glycemic control goal (100–180 mg/dL) by day 2 (*p* = 0.3). However, on the seventh or last day of basal-bolus treatment, whichever came first, 36.36% of patients receiving insulin detemir (*n* = 88) achieved the blood glucose reading goal compared to 52.00% in patients receiving insulin glargine (*n* = 100) (*p* = 0.03). This corresponded to an adjusted odds ratio of 2.12 (1.08 to 4.15), *p* = 0.03. The adjusting variables were provider type, whether the patient was hospitalized within 30 days prior and diagnosis of stroke. The mean blood glucose readings for the insulin glargine and the insulin detemir groups while on basal-bolus therapy were 200 mg/dL and 215 mg/dL, respectively (*p* = 0.05). The total number of blood glucose readings less than 70 mg/dL and less than 45 mg/dL was very low and there were no differences in number of episodes with hypoglycemia between the two groups. **CONCLUSION:** There was not a statistical difference between the two groups at 2 days, however there was on the seventh day or the last day of basal-bolus treatment. There were nonsignificant hypoglycemia events between basal insulin groups and the results for the last or seventh day of treatment may not be clinically significant in practice.

## 1. Introduction

Studies have shown that hyperglycemia is present in approximately 40% of patients at the time of hospital admission [1]. This is significant because hyperglycemia in hospitalized patients is often associated with adverse clinical outcomes, such as increased rates of morbidity, wound infections, length of stay, and mortality [1,2,3,4]. Hyperglycemia in the hospital setting not only occurs in those with diabetes, but also in patients with an acute illness due to stress who are non-diabetic patients [3].

In addition to managing hyperglycemia, hypoglycemia is a concern in hospitalized patients. Unnecessary morbidity and mortality can be the result of under-treatment or over-treatment of hyperglycemia [3]. The American Association of Clinical Endocrinologists (AACE) and the American Diabetes Association (ADA) consensus statement on inpatient glycemic control defines severe hypoglycemia as a blood glucose level less than 40 mg/dL, hypoglycemia as a blood glucose level less than 70 mg/dL, and hyperglycemia as a blood glucose level greater than 140 mg/dL [3,4]. The inpatient glucose target range for non-pregnant, noncritical ill adults with hyperglycemia is <140 mg/dL pre-meal and <180 mg/dL random, provided the targets can be achieved safely [3,5]. 

Insulin is the recommended pharmacotherapy for glucose management in the hospitalized patient [5]. The recommended glycemic management regimen consists of both basal and bolus insulins administered subcutaneously at scheduled intervals. Basal insulin refers to intermediate or long-acting insulin given once or twice daily, and bolus insulin consists of rapid or short-acting insulin administered before meals or every four hours [6]. A traditional insulin sliding scale algorithm with rapid or short-acting insulin scheduled as needed, also known as correctional insulin, is suggested for treatment of blood glucose levels above the target goal range [6]. Due to known spikes in blood glucose levels in acutely ill patients with or without known diabetes, basal-bolus treatment regimen is recommended for non-critical ill patients’ hyperglycemia management, as it most closely mimics the body’s normal patterns of insulin secretion [2]. In one study of type 2 diabetic non-critically ill patients, the basal-bolus regimen had greater improvement in glycemic control compared to pre-mixed insulin and sliding scale insulin regimens [7]. Due to the pharmacokinetic and pharmacodynamic predictability, rapid-acting insulin analogs, such as lispro, aspart, or glulisine, are preferred to regular insulin [5,8]. Long-acting insulin analogs, such as glargine or detemir, are superior to the intermediate-acting insulin neutral protamine hagedorn (NPH) due to the longer duration of action and peakless pharmacokinetic profile [2,8]. A Cochrane review analysis showed no clinical difference in the safety and efficacy for insulin glargine and insulin detemir in the treatment for type 2 diabetes [9]. Nevertheless, insulin detemir was frequently injected in a higher dose twice a day while insulin glargine was administered once a day in order to reach the same glycemic control [9]. 

In a crossover, randomized study comparing insulin detemir and insulin glargine administered once a day in hospitalized patients who had type 2 diabetes, there was not a significant difference in the glucose control for 55 patients [10]. The authors suggested differences in action may exist between the basal insulins, however more studies should be performed [10]. Since there is limited research comparing insulin glargine to detemir in the hospital setting, this study was conducted. 

## 2. Materials and Methods

A retrospective study was conducted at two acute care community hospitals in the United States at Medical Center Arlington in Arlington, Texas and at Medical Center Plano in Plano, Texas. Patients’ oral diabetic medications and/or insulin was discontinued and changed to the basal-bolus regimen once the protocol was initiated by the provider e.g., physician or licensed independent practitioner. All patients at the acute care hospital where the patients received insulin detemir as the long-acting agent in the basal-bolus protocol for more than 48 h between July 2012 and August 2013 were included in the analysis. At the acute care hospital where patients received insulin glargine as the long-acting agent, every 25th patient on the basal-bolus protocol for more than 48 h between January 2012 and September 2012 was included in the analysis. If the 25th patient was not on the protocol for more than 48 h, the next patient was analyzed. Due to the greater number of patients receiving insulin glargine versus insulin detemir, not all of the patients were included in the analysis. Patients who were pregnant, post-partum, on intravenous (IV) insulin at the hospital, or under 18 years of age were excluded from the study. The objective of the study was to determine whether insulin detemir was similar to insulin glargine in controlling hyperglycemia in the adult hospitalized patient on a basal-bolus treatment regimen. Other endpoints of the study included antihyperglycemics at discharge, discharge diagnosis, and the number of injections that patients received. 

Patient characteristics were collected upon admission. Comorbidities were also recorded at the time of admission. The basal-bolus characteristics considered were blood sugar on admission and prior to starting the basal-bolus protocol, HgA1c level, total number of injections, total units of insulin, average blood glucose during the basal-bolus treatment, number of long-acting insulin units, number of short-acting insulin units, number and percentage of blood glucose readings greater than 180 mg/dL, less than 70 mg/dL, and less than 45 mg/dL during the first seven days of basal-bolus treatment. Only data for the first seven days of basal-bolus treatments were considered for analysis. Statistically significant outcomes (*p* < 0.05) were adjusted for patient characteristics and comorbidities that were found to be significantly different between the two hospitals. For the unadjusted and adjusted comparison of outcomes between patients receiving insulin detemir and insulin glargine, the odds ratio and estimate ratio are obtained from models that adjust for provider type, whether the patient was hospitalized 30 days prior, and for stroke. 

The study evaluated patients from two facilities who were initiated on a basal-bolus subcutaneous insulin protocol regimen. One facility administered insulin detemir as their long-acting insulin and one facility administered insulin glargine as their long-acting insulin. The basal-bolus regimen consisted of three components: basal insulin (daily or twice daily long-acting insulin injections), bolus or prandial insulin (scheduled rapid-acting insulin injections), and corrective insulin (rapid-acting insulin injections). (See Table 1 for the corrective algorithm.) 

The basal-bolus protocol was developed by providers, based upon the literature, and initiated at each facility. The initial total daily insulin dose was determined by the physician and varied by patient condition: 0.3 units/kg for patients on hemodialysis or who were sensitive to insulin; 0.4 units/kg for typical patients receiving the standard regimen; 0.5 units/kg for overweight patients as defined by the provider; or 0.6 units/kg for patients resistant to insulin. The physician was permitted to tailor the total daily dose if he or she deemed a higher or lower dose was needed. 

The physician could select a basal-bolus protocol regimen from three different scenarios. Scenario one was designed for patients either eating normally or receiving bolus tube feeds, scenario two was designed for patients receiving continuous tube feeding or parental nutrition, and scenario three was designed for patients who were not receiving anything orally (NPO) or nearly NPO (taking clear liquids only) and were not receiving continuous tube feedings or parental nutrition. For scenario one, patients received fifty percent of the total daily dose as basal insulin, fifty percent as bolus insulin divided equally before each meal, and a corrective insulin before each meal and at bedtime as needed per the appropriate algorithm. For scenario two, patients received fifty percent of the total daily dose as basal insulin, fifty percent as bolus insulin divided equally and administered every 4 h, and corrective insulin every 4 h as needed per the appropriate algorithm. For scenario three, patients received fifty percent of the total daily dose as basal insulin and corrective insulin every 4 h as needed per the appropriate algorithm. Corrective insulin was administered when blood glucose values were above the target. For scenario three, a continuous infusion of dextrose 5% and 0.45% sodium chloride was recommended. Insulin aspart was utilized in both study groups for the bolus and/or corrective insulin doses. For the patients receiving insulin detemir as the basal dose, the dose was divided into two equal doses when desired units were greater than or equal to thirty.

As part of the basal-bolus protocol for patients receiving insulin detemir and insulin glargine, the total daily dose was evaluated daily; based upon the glycemic control of the previous twenty-four hours, the regimen was either adjusted or continued. If at any time a glucose value was less than 90 mg/dL, even if all scheduled doses were not given, the total daily dose was decreased by twenty percent. All increases were made only if all scheduled doses were given. The total daily insulin dose was increased by twenty percent if any one glucose level was greater than 200 mg/dL or by thirty percent if two or more glucose levels were greater than 200 mg/dL. For the increase, fifty percent of the total daily dose was administered as basal insulin (scenarios one, two, and three) and fifty percent as bolus insulin (scenario one and two). If the blood glucose at any time was less than 70 mg/dL, a hypoglycemic algorithm was initiated which consisted of the following: 15 g of carbohydrates orally if able to swallow, 25 g of dextrose intravenously if unable to swallow, or 1 mg of glucagon intramuscularly if the patient did not have intravenous access. In all patients, the provider was notified and a repeat blood glucose test was completed 15 min after treatment. 

Institutional Review Board approval was obtained and patient’s information was de-identified. 

The primary outcome of this study was the percentage of patients who reached at least one blood glucose reading goal of 100–180 mg/dL on the seventh or last day of treatment, whichever came first. The blood glucose was drawn by either point of care testing and/or venous blood draw. Blood glucose readings were performed at a minimum prior to each administration of insulin and documented in the medical record. Secondary outcomes were number of days to achieve the first reading goal, average blood glucose reading during the basal-bolus treatment, length of basal-bolus treatment, hospital length of stay, hypoglycemia events, discharge disposition, antihyperglycemics at discharge and discharge diagnosis.

Continuous variables were summarized by means and standard deviation and also by median and interquartile range (IQR). Categorical variables were summarized using count and percentages. Demographics, comorbidities and basal-bolus characteristics were compared between patients receiving insulin detemir and patients receiving insulin glargine. The Wilcoxon rank sum test for continuous variables and Chi-square test for categorical variables were also used.

The two treatment groups were compared in outcome variables described above. The initial comparison was performed using Wilcoxon rank sum test for continuous outcomes (hospital length of stay, average blood glucose reading on basal-bolus therapy and length of basal-bolus treatment), chi-square test for categorical outcomes (discharge disposition and discharge blood sugar medication), and log-rank test for days to achieve first blood glucose reading goal. Statistically significant outcomes were compared in multivariable models. The models were adjusted for patient characteristics and comorbidities that were found significantly different among the two hospitals. Log-linear models were used to compare continuous outcomes and logistic regression was used to compare categorical outcomes.

## 3. Results

There was a total of 188 patients included in the study at two separate hospitals. The baseline characteristics were similar between the patients receiving insulin detemir and the patients receiving insulin glargine. (See Table 2.) Most patients were under the care of a hospitalist as the provider (100% for patients receiving insulin detemir; 69% for patients receiving insulin glargine). At the time of admission, very few patients were on medications that increased blood glucose (approximately 15% at both hospitals). There were more patients on insulin detemir who were hospitalized within the last 30 days prior to admission (PTA) (*p* = 0.003) than the patients receiving insulin glargine. The comorbidity in both the patients receiving insulin detemir and the patients receiving insulin glargine were similar except for the number of patients with a diagnosis of stroke. (See Table 3.)

High median admission HgA1c levels 9.31% (SD 2.42) for patients receiving insulin detemir; 8.06% (SD 2.17) for patients receiving insulin glargine (*p* = 0.0007) were noted. Median admission blood glucose levels 244 mg/dL (IQR 166–368 mg/dL) for patients receiving insulin detemir and 226 mg/dL (IQR 172–314 mg/dL) for patients receiving insulin glargine (*p* = 0.61) were present even though most were known to have diabetes mellitus. During admission, the median number of injections for patients receiving insulin detemir (18; IQR 13–30) and units of insulin (206 units; IQR 153–440) were fewer than patients receiving insulin glargine (24; IQR 16–29 with 275 units; IQR 167–458) respectively, which was not statistically significant. For patients receiving insulin detemir, overall median blood glucose levels (211 mg/dL; IQR 172–247) were higher than patients receiving insulin glargine (190 mg/dL; IQR 154–244) (*p* = 0.05). Patients receiving insulin glargine had fewer blood glucose readings (53%) above 180 mg/dL compared to patients receiving insulin detemir (64%) (*p* = 0.04). Both the patients receiving insulin detemir and the patients receiving insulin glargine had less than 2% of blood glucose readings less than 70 mg/dL. (See Table 4.) 

In both groups, after 2 days, 50% had achieved the blood glucose reading goal (*p* = 0.26). The length of basal-bolus treatment was about 6 days on average for both the patients receiving insulin detemir and the patients receiving insulin glargine, and patients receiving insulin glargine had a median longer hospital stay of 10 days versus 7.5 days for patients receiving insulin detemir. After adjusting for different patient characteristics, it was found that patients receiving insulin glargine had a 43% longer length of stay than patients receiving insulin detemir (estimate ratio: 1.43:1.10–1.85; 95% CI).

**Figure 1:**
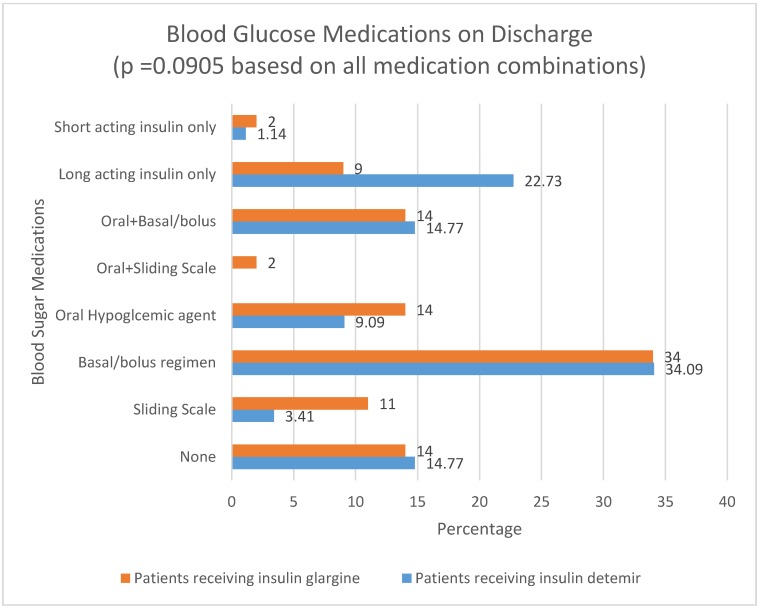
Blood glucose medications on discharge.

On the seventh or last day of basal-bolus treatment, whichever occurred first, 36% of patients receiving insulin detemir achieved the blood glucose reading goal compared to 52% of patients receiving insulin glargine (*p* = 0.03). This corresponded to an adjusted odds ratio of 2.12 (1.08 to 4.15). The adjusting variables were provider type, whether the patient was hospitalized within 30 days prior, and diagnosis of stroke. For unadjusted and adjusted comparison of outcomes between patients receiving insulin detemir and insulin glargine, see Table 5. Patients receiving insulin detemir had a higher discharged-to-home percentage (68.18% vs. 54.00%; *p* = 0.047), and more patients receiving insulin glargine were discharged to a Nursing Home or other long-term acute care facility (31.00% vs. 15.91%) (*p* = 0.02). Neither outcome was statistically significant in adjusted comparisons. The type of blood sugar medication on which a patient was discharged is noted in Figure 1. Patients receiving insulin glargine (7%) had a higher mortality rate than patients receiving insulin detemir (1.14%) (*p* = 0.047).

## 4. Discussion

The study authors’ objective was to determine whether insulin detemir and insulin glargine were similar if used in two similar size and type hospitals in controlling hyperglycemia in the adult hospitalized patients on basal-bolus treatment regimens. A high number of patients from both groups receiving insulin detemir and insulin glargine were known diabetics and had high admission blood glucose and HgA1c levels. Overall, a high percentage had heart failure as a comorbidity, as well as Chronic Obstructive Pulmonary Disease (COPD), malignancy, and had been hospitalized within the previous 30 days. This may have indicated that these patients were quite ill when they were admitted to the hospital and had many long-term illnesses.

Although not statistically significant, the results showed that patients receiving insulin glargine received more units of insulin and injections than patients receiving insulin detemir. It is unknown as to why more units and injections were administered, however this could explain why more of these patients reached their target glycemic goal. Possibilities could include lack of administration of insulin or adherence to the protocol. In contrast, a crossover randomized study in type 1 diabetes, comparing insulin glargine to insulin detemir showed similar blood glucose control, however there was an increase in the number of daily injections and higher doses with detemir [11]. Half of the number of patients receiving insulin detemir and insulin glargine achieved target blood glucose reading (100–180 mg/dL) as defined by the protocol in about two days. For the insulin detemir group, the decrease in goal range from two days to either the seventh day or last day of discharge by 14% may have been due to a limited number of readings on the last day when the patients were discharged. The duration of basal-bolus therapy was similar, but the overall mean blood glucose was lower in patients receiving insulin glargine. 

A greater percentage of patients receiving insulin glargine were discharged to a long-term acute care hospital or a nursing home (31% vs. 16%). Hospital length of stay was significantly less for patients receiving insulin detemir (7.5 days) than patients receiving insulin glargine (10 days), and mortality was significantly lower for patients receiving insulin detemir (1) than patients receiving insulin glargine (7). This difference may have been due to other factors including acuity. 

The two hospitals in the study are located in the same geographic area but have different staff and providers. The same basal-bolus insulin orders were used in the patients receiving insulin detemir and the patients receiving insulin glargine; however, daily adjustments were required and may have been completed differently in each facility depending upon the healthcare provider caring for the patient. In addition, education of providers on use of basal-bolus therapy was variable among treatment groups. Some possible reasons for non-adherence to the protocol include patient being off the unit, patient refusing medication, NPO, or the nurse not administering the prescribed dose. 

Basal-bolus dosing of insulin in acutely ill hospitalized patients is a complex therapy for clinicians and requires careful patient monitoring. Monitoring of the patients included not only hyperglycemica but also hypoglycemia which was not significant. In the treatment of diabetes, long-acting basal insulins such as insulin detemir and insulin glargine are a necessary component of the insulin treatment regimen [12]. Comprehensive education of nurses, pharmacists, dietitians, and medical providers is essential to patient safety and success of basal-bolus therapy for managing high blood glucose. As shown in this retrospective review, patients who are known diabetics may not be managed with insulin therapy outside of the hospital, have complex medical conditions, and may be admitted to the hospital with high HgA1c and blood glucose levels. Achieving the target blood glucose reading with basal-bolus therapy provides the steadiest glycemic management to the patient. The concept of basal-bolus therapy with daily adjustments requires adherence to the protocol and careful monitoring in order to avoid hypoglycemic episodes, as well as persistent hyperglycemia. 

This was a retrospective study, whereby data were truncated to seven days. This data truncation may have resulted in different readings of blood glucose levels obtained. Other limitations include lack of power analysis; incomplete data and omission of administration of insulin administered to the patient during admission; selection bias with every 25th patient in the insulin glargine patient group and with date ranges; short-acting insulin not differentiated from prandial and correctional doses; and not monitoring adherence to the protocol. In addition, one confounder could be the different patient scenarios that the provider could choose based upon the status of the patient. Attending provider, monitoring of patients, daily adjustments, and education of staff may all have contributed to differences in data. Even though the education varied by site, education was provided to nursing and providers when needed. Strict adherence to the basal-bolus order set was hospital- and provider-dependent and not concurrently monitored by researchers. 

Since the results may not be clinically significant in practice, each individual facility may evaluate which basal insulin would best fit their needs. The Pharmacy and Therapeutics Committee could review the long-acting insulins for not only effectiveness but also medication safety, adverse reactions, pharmacokinetics, and mechanism of action. By reviewing all of these factors, a hospital may determine which medication they would add to formulary. 

## 5. Conclusions

Even though the last or seventh day of treatment was statistically significant, the results may not be clinically significant in practice. The patients receiving insulin glargine were administered more insulin injections and total units of insulin without increased episodes of hypoglycemia, which may have impacted the blood sugar readings. The hospital length of stay was longer for the patients on insulin glargine and a larger percentage of patients had a history of stroke, which could have affected mortality, length of stay, and discharge. Other patient factors may have contributed to the differences. Further larger prospective randomized studies are needed for more comparisons of insulin detemir to insulin glargine.

## Figures and Tables

**Table 1:** Corrective bolus insulin algorithm for the basal-bolus treatment regimen.

Insulin Low Dose Algorithm (for Patients Requiring Less Than or Equal to 40 Units of Insulin per Day)			Insulin Medium Dose Algorithm (for Patients Requiring 41–80 Units of Insulin per Day)			Insulin High Dose Algorithm (for Patients Requiring Greater than 80 Units of Insulin per Day)		
Prandial Blood Sugar	Correctional Dose (Units)	Bedtime Insulin Dose Only (Units)	Prandial Blood Sugar	Correctional Dose (Units)	Bedtime Insulin Dose Only (Units)	Prandial Blood Sugar	Correctional Dose (Units)	Bedtime Insulin Dose Only (Units)
151–199	1	0	151–199	2	0	151–199	3	0
200–249	2	0	200–249	4	1	200–249	5	2
250–299	3	1	250–299	5	2	250–299	7	3
300–349	4	2	300–349	7	3	300–349	10	4
350 or Greater	6	3	350 or Greater	8	5	350 or Greater	12	5

**Table 2:** Patient characteristics at admission.

Variable	Patients Receiving Insulin Detemir (n = 88)	Patients Receiving Insulin Glargine (n = 100)	*p*-Value
**Age**			0.7758
Mean (SD)	67.1 (23.0)	62.9 (13.6)	
Median (IQR)	62.5 (53.0–78.0)	64.0 (54.0–73.0)	
**Weight (kg)**			0.6004
Mean (SD)	90.0 (28.0)	92.5 (29.5)	
Median (IQR)	85.5 (71.0–103.5)	88.0 (71.7–105.0)	
**Gender**				
Female, n (%)	44 (50.00)	51 (51.00)	0.5639
**Ethnicity**			0.1823
Caucasian, n (%)	48 (54.55)	70 (70.00)	
African-American, n (%)	16 (18.18)	14 (14.00)	
Hispanic, n (%)	14 (15.91)	10 (10.00)	
Asian, n (%)	5 (5.68)	5 (5.00)	
Other, n (%)	5 (5.68)	1 (1.00)	
**Admitting diagnosis and code**			0.0552
ID, n (%)	11 (12.50)	16 (16.00)	
CVA, n (%)	2 (2.27)	9 (9.00)	
CVD, n (%)	13 (14.77)	20 (20.00)	
Surgical, n (%)	2 (2.27)	6 (6.00)	
Medical, n (%)	60 (68.18)	49 (48.00)	
**Provider type**			< 0.0001
Hospitalist, n (%)	88 (100.00)	69 (69.00)	
Internist, n (%)	0 (0.00)	28 (28.00)	
Surgeon, n (%)	0 (0.00)	3 (3.00)	
**Patient on meds that increased Blood Sugar PTA**			0.6857
Yes, n (%)	14 (15.91)	14 (14.00)	
**Patient on insulin or oral hypoglycemic PTA**			0.0724
None, n (%)	19 (21.59)	17 (17.00)	
Insulin, n (%)	33 (37.08)	43 (43.00)	
Hypoglycemic agent, n (%)	24 (27.27)	16 (16.00)	
Both, n (%)	11 (12.5)	24 (24.00)	
Missing, n (%)	1 (1.14)	0 (0.00)	
**Patient hospitalized 30 days prior to this admission**			0.0029
No, n (%)	53 (60.23)	75 (75.00)	
Yes, n (%)	24 (27.27)	22 (22.00)	
Unknown, n (%)	11 (12.50)	1 (1.00)	
Missing, n (%)	0 (0.00)	2 (2.00)	
**Nutrition status**			0.1620
NPO, n (%)	9 (10.23)	8 (8.00)	
Eating regular meals, n (%)	75 (85.23)	78 (78.00)	
TF/TPN, n (%)	4 (4.55)	13 (13.00)	
Clear Liquid, n (%)	0 (0.00)	1 (1.00)	
**HgA1c on admission (%)**			0.0007
Patients with complete data	68	96	
Mean (SD)	9.31 (2.42)	8.06 (2.17)	
Median (IQR)	9.15 (7.55–11.0)	7.75 (6.40–9.55)	

SD = Standard deviation; IQR = Interquartile range; ID = Infectious Disease; CVA = Cerebrovascular Accident; CVD = Cardiovascular Disease; PTA = Prior to Admission; NPO = Nothing orally; TF = Tube feeding; TPN = Total Parenteral Nutrition; HgA1c = Hemoglobin A1c.

**Table 3:** Patient comorbidities.

Variable	Patients Receiving Insulin Detemir (n = 88)	Patients Receiving Insulin Glargine (n = 100)	*p*-Value
Diabetes Mellitus (Type 1 or Type 2), n (%)	81 (92.05)	90 (90.00)	0.4243
Heart Failure, n (%)	29 (32.95)	22 (22.00)	0.0919
COPD, n (%)	16 (18.18)	17 (17.00)	0.8317
Hepatic Dysfunction, n (%)	5 (5.68)	3 (3.00)	0.3545
Dialysis, n (%)	4 (4.55)	7 (7.00)	0.4743
Transplanted organ, n (%)	2 (2.27)	1 (1.00)	0.4807
Malignancy (cancer), n (%)	11 (12.50)	12 (12.00)	0.9169
HIV, n (%)	1 (1.14)	1 (1.00)	0.9275
Stroke, n (%)	9 (10.23)	25 (25.00)	0.0301

COPD = Chronic Obstructive Pulmonary Disease; HIV = Human Immunodeficiency Virus.

**Table 4:** Basal-bolus (BB) treatment characteristics and glycemic control results based on basal-bolus treatment.

Variable	Patients Receiving Insulin Detemir (*n* = 88)	Patients Receiving Insulin Glargine (*n* = 100)	*p*-Value
**Blood Sugar (BS) on admission (mg/dL)**			
Mean (SD)	286 (170)	267 (137)	
Median (IQR)	244 (166–368)	226 (172–314)	0.6079
**Last BS prior to starting BB (mg/dL)**			
Mean (SD)	261 (96)	239 (94)	
Median (IQR)	258 (186–308)	226 (174–285)	0.0928
**Total number of injections**			
Patients with complete data	87	100	
Mean (SD)	26 (25)	25 (15)	
Median (IQR)	18 (13–30)	24 (16–29)	0.1092
**Total units of insulin**			
Patients with complete data	87	100	
Mean (SD)	393 (521)	372 (363)	
Median (IQR)	206 (153–440)	275 (167–458)	0.3913
**Mean Blood glucose while on BB (mg/dL)**			
Patients with complete data	87	100	
Mean (SD)	215 (57)	200 (58)	
Median (IQR)	211 (172–247)	190 (154–244)	0.0500
**Long-acting units**			
Mean (SD)	132 (98)	151 (103)	
Median (IQR)	98 (75–179)	123 (80–177)	0.1703
**Short-acting units**			
Mean (SD)	155 (121)	180 (136)	
Median (IQR)	114 (79–203)	144 (76–248)	0.2743
**All Blood glucose readings >180 mg/dL**			
Mean (SD)	12 (6)	11 (7)	
Median (IQR)	11 (8–15)	10 (6–15)	0.1990
Percentage			
Mean (SD)	61% (26%)	52% (29%)	
Median (IQR)	64% (43%–80%)	53% (31%–74%)	0.0425
**All blood glucose readings <70 mg/dL**			
Mean (SD)	0.32 (0.74)	0.31 (0.68)	
Median (IQR)	0 (0–0)	0 (0–0)	0.8893
Percentage			
Mean (SD)	1.5% (3.4%)	1.3% (3.0%)	
Median (IQR)	0% (0%–0%)	0% (0%–0%)	0.9970
**All blood glucose readings <45 mg/dL**			
Mean (SD)	0.02 (0.15)	0.03 (0.17)	
Median (IQR)	0 (0–0)	0 (0–0)	0.7578
Percentage			
Mean (SD)	0.1% (0.8%)	0.1% (0.8%)	
Median (IQR)	0% (0%–0%)	0% (0%–0%)	0.7578
**Days of BB therapy**			
Mean (SD)	6.66 (4.84)	6.18 (3.39)	
Median (IQR)	5.00 (4.00–8.00)	6.00 (4.00–7.00)	0.7947

SD = Standard deviation; IQR = Interquartile range; BS = Blood sugar; BB = Basal-bolus.

**Table 5:** Unadjusted and adjusted comparison of outcomes between patients receiving insulin detemir and insulin glargine.

Variable	Patients Receiving Insulin Detemir (*n* = 88)	Patients Receiving Insulin Glargine (*n* = 100)	*p*-Value
**Hospital length of stay**			
Mean (SD)	9.67 (7.54)	13.4 (9.68)	
Median (IQR)	7.50 (4.00–12.0)	10.0 (6.00–17.0)	0.0018
Estimate Ratio (95% CI)	Reference	1.43 (1.10–1.85)	
**Discharge disposition**			
Home			
n (%)	60 (68.18)	54 (54.00)	0.0470
Odds Ratio (95% CI)	Reference	0.62 (0.31–1.24)	
Hospital, n (%)	1 (1.14)	4 (4.00)	0.2234
Nursing Home/LTC			
n (%)	14 (15.91)	31 (31.00)	0.0155
Odds Ratio (95% CI)	Reference	2.26 (0.97–5.30)	
Other			
n (%)	12 (13.64)	4 (4.00)	0.0181
Odds Ratio (95% CI)	Reference	0.44 (0.14-1.39)	
Expired			
n (%)	1 (1.14)	7 (7.00)	0.0469
Odds Ratio (95% CI)	Reference	3.74 (0.46–30.4)	
**Within the reading goal on the last or seventh day of treatment, n (%)**			
n (%)	32 (36.36)	52 (52.00)	0.0314
Odds Ratio (95% CI)	Reference	2.12 (1.08–4.15)	
**Days to achieve the first reading goal**			
Median (IQR)	2 (0–6)	2 (0–5)	0.2589
Hazard Ratio (95% CI)	Reference	0.97 (0.69–1.36)	

SD = Standard deviation; IQR = Interquartile range; CI = Confidence Interval; LTC = Long-Term Care.
